# Evaluating the Impact of Intervention Strategies on the First Wave and Predicting the Second Wave of COVID-19 in Thailand: A Mathematical Modeling Study

**DOI:** 10.3390/biology10020080

**Published:** 2021-01-22

**Authors:** Wiriya Mahikul, Palang Chotsiri, Kritchavat Ploddi, Wirichada Pan-ngum

**Affiliations:** 1 Faculty of Medicine and Public Health, HRH Princess Chulabhorn College of Medical Science, Chulabhorn Royal Academy, Bangkok 10210, Thailand; wiriya.mah@cra.ac.th; 2 Department of Fundamentals of Public Health, Faculty of Public Health, Burapha University, Chonburi 20131, Thailand; 3 Mahidol-Oxford Tropical Medicine Research Unit, Faculty of Tropical Medicine, Mahidol University, Bangkok 10400, Thailand; palang@tropmedres.ac; 4 Division of Epidemiology, Department of Disease Control, Ministry of Public Health, Nonthaburi 11000, Thailand; dr.kritchavat@gmail.com; 5 Department of Tropical Hygiene, Faculty of Tropical Medicine, Mahidol University, Bangkok 10400, Thailand

**Keywords:** mathematical modelling, coronavirus disease 2019 (COVID-19), Bayesian approach, intervention, prediction

## Abstract

**Simple Summary:**

The world is currently experiencing the COVID-19 pandemic, consequently, we developed a compartmental model to describe the transmission dynamic of the disease, which can reproduce the incidence of COVID-19 first wave in Thailand. Screening incoming visitors, contact tracing, and case investigation were introduced from the beginning of the pandemic, while the rapid reduction of new cases was a result of the declaration of emergency decree in March 2020. The validated model was used to quantify the impacts of these intervention strategies. The model predicted that the daily reported incidence would have reached zero by the end of June if the on-going non-pharmaceutical interventions (NPIs) were strictly and widely implemented. Our study provides a better understanding of the first wave COVID-19 pandemic and the impacts of government interventions in a Thai setting, where data were still limited. The model further explored the use of these available interventions in the scenario analysis to control the emergence of a second wave of COVID-19 in Thailand. Continued good practice of social distancing to minimize the contact rates is still necessary while the vaccines are not fully available to all populations.

**Abstract:**

Coronavirus disease 2019 (COVID-19) has spread rapidly worldwide. This study aimed to assess and predict the incidence of COVID-19 in Thailand, including the preparation and evaluation of intervention strategies. An SEIR (susceptible, exposed, infected, recovered) model was implemented with model parameters estimated using the Bayesian approach. The model’s projections showed that the highest daily reported incidence of COVID-19 would be approximately 140 cases (95% credible interval, CrI: 83–170 cases) by the end of March 2020. After Thailand declared an emergency decree, the numbers of new cases and case fatalities decreased, with no new imported cases. According to the model’s predictions, the incidence would be zero at the end of June if non-pharmaceutical interventions (NPIs) were strictly and widely implemented. These stringent NPIs reduced the effective reproductive number (Rt) to 0.73 per day (95% CrI: 0.53–0.93) during April and May. Sensitivity analysis showed that contact rate, hand washing, and face mask wearing effectiveness were the parameters that most influenced the number of reported daily new cases. Our evaluation shows that Thailand’s intervention strategies have been highly effective in mitigating disease propagation. Continuing with these strict disease prevention behaviors could minimize the risk of a new COVID-19 outbreak in Thailand.

## 1. Introduction

Coronavirus disease 2019 (COVID-19) is an emerging infectious disease caused by severe acute respiratory syndrome coronavirus 2 (SARS-CoV-2), which continues to spread globally. The first case report was made on 31 December 2019 in China. On 31 August 2020, the World Health Organization (WHO) reported that numbers of reported cases and deaths worldwide (213 countries) had reached 25,383,993 confirmed cases and 850,588 confirmed deaths [1]. In Thailand, the reported number of confirmed cases was 3412, with 58 deaths [2]. Thailand was the first country outside of China to confirm and report a case of COVID-19, on 12 January 2020. Two major transmission clusters, related to a nightclub and a boxing stadium and comprising a total of 188 cases, were reported on 22 March 2020 [3]. Several countries have implemented non-pharmaceutical interventions (NPIs) to mitigate the spread of the disease; these NPIs include physical distancing, face masks, and eye protection [4,5,6]. Since 3 January 2020, the Thai Ministry of Public Health has implemented public health and medical measures by screening incoming visitors from affected areas; carrying out epidemiological investigations, including case investigation and contact tracing; and developing clinical practice guidelines [7,8]. The Thai government then announced an emergency decree and a nationwide curfew on March 26 and 3 April 2020, respectively, which included banning people from leaving their residence during night time, the prohibition of public gatherings, restrictions on international travel, and restrictions on people moving between provinces. The government also suggested that face masks be worn in public and that people maintain social distancing, work from home, and remain inside their residence from 10 p.m. to 4 a.m. After these policies had been implemented, the number of daily reported cases of COVID-19 decreased [9]. A 14-day state or alternative quarantine for both foreigners and Thai nationals entering Thailand was also strictly enforced [2]. To prevent a second wave of transmission, these strict policies should be continued [9]. However, these measures have had to be eased for socioeconomic reasons. Businesses considered to be at low risk for transmission of COVID-19 (such as markets, food shops, street stalls, and hair salons) were allowed to reopen on 3 May 2020. After no locally transmitted cases had been reported for more than a month, high-risk businesses, including night clubs and massage parlors, were allowed to reopen on 1 July 2020 [10]. However, the Thai public were advised to follow the ‘new normal’ guidelines, such as maintaining social distancing, wearing a face mask, and working from home.

Several mathematical modeling studies have been conducted to predict the incidence of COVID-19, evaluate the impact of each intervention strategy, and assist the government in policymaking to mitigate the spread of disease. A susceptible-exposed-infectious-recovered (SEIR) model was applied to predict the number of COVID-19 cases and estimate the basic reproductive number (R0) in Wuhan, China [11]. The R0 was initially predicted to be 2.68 (95% credible interval (CrI): 2.47–2.86), and the epidemic doubling time was 6.4 days (95% CrI: 5.8–7.1 days). The estimated R0 for COVID-19 in Thailand was estimated to be 3.75 (95% CI: 2.23–5.90) [12], while the effective reproduction number (Rt), equivalent to a time-varying R0 dependent of the number of susceptible individuals, was reduced to less than 1 in the period after the announcement of the emergency decree [12,13]. An SEIR model was applied to examine the impact of social distancing and working from home. These interventions during the period when measures were relaxed were predicted to reduce the median number of infections by 92% by the middle of 2020 compared with the number if no interventions were made [14]. Other modeling work suggested that travel control measures were one of the most effective strategies, reducing Rt from 2.35 (95% CrI: 1.15–4.77) to 1.05 (95% CrI: 0.41–2.39) [15]. A proactive policy of testing and contact tracing can help keep the disease within the capacity of a healthcare system during the relaxation of social distancing interventions [16,17,18]. However, a second wave of infections is likely to occur with a magnitude 2.0–2.3-times that of the first wave when the restrictions are lifted in the UK [19]. To prevent the impending second wave, an increasing amount of proactive testing and contact tracing, with isolation of confirmed cases, has been recommended [19]. In the present study, we constructed a compartmental model to evaluate the impact of interventions such as social distancing, working from home, hand washing, wearing a face mask, and quarantine, in the Thai context. We further used the model to predict the possible incidence of a new wave of COVID-19 in Thailand.

## 2. Materials and Methods

### 2.1. Model Structure

**Figure 1:**
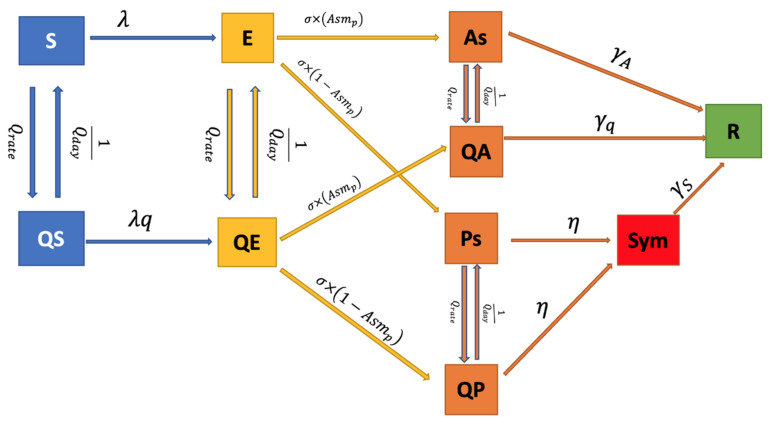
Schematic representation of the COVID-19 dynamic model.

We constructed a compartmental model (a susceptible, exposed, infected, recovered (SEIR) model). We then divided the population of Thailand into ten health compartments including 5 disease stages without and 5 stages with quarantine: susceptible (S), exposed (E), asymptomatic infection (As), pre-symptomatic infection (Ps), recovered (R), quarantine susceptible (QS), quarantine exposed (QE), quarantine asymptomatic infection (QA), quarantine pre-symptomatic clinical (QP), and symptomatic infection (Sym) (illustrated in Figure 1). As some individuals may go the entire course of infection and never experience symptoms (as defined as asymptomatic individuals (As and QA)), other individuals who will later develop symptoms are defined as being pre-symptomatic (Ps and QP) [20]. The symptomatic stage is defined for those who developed symptoms and thus quarantined. The population was stratified by age into eight groups as follows: 0–4, 5–14, 15–24, 25–34, 35–44, 45–54, 55–64, and more than 64 years of age. The population in each age group followed the actual population structure of Thailand, obtained from the Population and Housing Census in 2020 [21]. All females aged between 15 and 54 years were considered to be capable of reproduction, with a fertility rate (*fr*), whereas the mortality rate was age-related [22]. We solved a large set of 10 × 8 ordinary differential equations (ODEs), capturing both population and disease dynamics, and estimated some parameters for the deterministic model, defined in the Supplementary Materials.

The daily reported cases (incidence) stratified by age and daily reported outcomes (recovery and death) stratified by age were provided by the Center for COVID-19 Situation Administration (CCSA) and the Department of Disease Control, Ministry of Public Health (MoPH), Thailand [2]. Key assumptions in the model were as follows: (1) an equivalent transmission rate between sexes, (2) the unavailability of an effective vaccine, (3) long-term immunity to reinfection among recovered cases, (4) a homogenous possibility of infection, (5) isolation of symptomatic patients to reduce future transmission, (6) no seasonal effect in the model, and (7) all high-risk contacts who stay in the same household as symptomatic cases (assuming an average of three people) with no symptoms were traced and quarantined.

### 2.2. Model Simulations

R software, version 3.2.3 (R Core Team, Vienna, Austria), was used to run the model and analyze its outputs, with the system’s dynamic differential equations solved using the deSolve package [23]. The initial parameter values were calculated from the population data and disease burden. A period of 12 months was executed, starting on 1 January 2020 and running to 1 January 2021. A certain degree of quarantine and isolation were included from the start, prior to the implementation of the government’s state of emergency decree, through contact tracing, case isolation and quarantining. The model was simulated by the interaction between population by age and the transmission of the disease.

### 2.3. Model Validation and Projection

Model fitting was carried out using the Markov Chain Monte Carlo (MCMC) method, implemented in the Bayesian Tools R package, defined in the Supplementary Materials [24]. The model was then run and fitted to the daily reported cases (incidence) by age and daily reported outcomes (recovery and death) by age, from 12 January 2020 to 17 June 2020 [2]. Two separate chains and three cores, each consisting of 50,000 iterations and a burn-in period of 12,500 iterations, were run in parallel to achieve a target acceptance rate of 0.2 (Supplementary Materials, Figure S1). Several measures were used to assess convergence, including the standard Gelmal-Rubin procedure [25] and target acceptance rates [26]. The median values and credible intervals are shown in the Supplementary Materials, Table S1. Five parameters were estimated, including a daily contact rate after Thailand declared its emergency decree and before the easing of each restriction phase, the infectivity rate of COVID-19, the daily mean number of infected migrants, the percentage of reported asymptomatic infections, and the case fatality proportion by age. The model was further used to project a second wave of COVID-19 in Thailand, sampling all parameters from the posterior chains. In addition, the effective reproductive number, Rt, was estimated and is presented in the Supplementary Materials, Figure S2.

**Figure 2:**
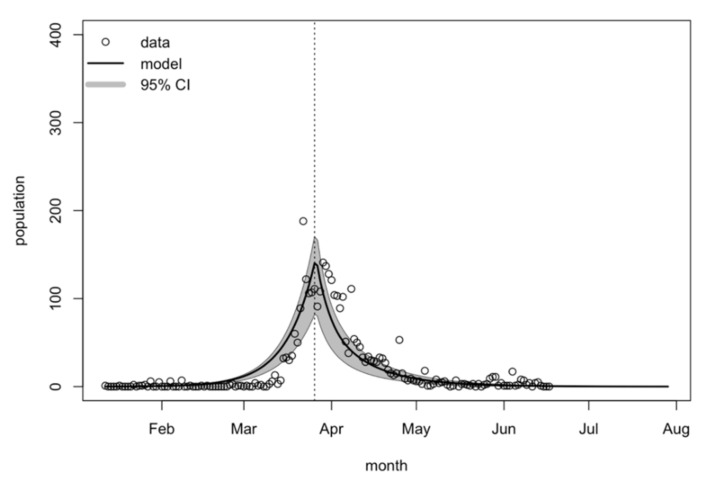
Reported COVID-19 confirmed cases and the model estimation with 95% CrI between January and August 2020 (vertical dotted line: the date the Thai government announced the emergency decree, on 26 March 2020).

Figure 2 shows the model fitting performance. Reported COVID-19 incidence cases per day were plotted against the model’s predicted incidence. The highest number of estimated reported incidence cases per day was about 140 (95% CrI: 83–170) at the end of March 2020. The Thai government announced the emergency decree on 26 March 2020, resulting in a decreasing number of new COVID-19 cases. The model estimated that the incidence would reach zero by the end of June if the current interventions were strictly implemented nationwide, including social distancing, working from home, hand washing, wearing masks, contact tracing, case isolation, and quarantining. The model’s estimations for recovered cases and deaths are shown in the Supplementary Materials, Figures S3 and S4, respectively. The highest estimated number of recovered cases per day was 83 (95% CrI: 28–122) in the middle of April 2020. The highest estimated number of deaths per day was 4 (95% CrI: 2–6) at the end of March 2020.

### 2.4. Sensitivity Analysis

Sensitivity analyses were performed on the uncertainties around contact rates during the emergency decree, the percentage of all symptomatic infections that were reported, the effectiveness of hand washing and wearing of face masks, quarantine effectiveness, quarantine coverage, and the mean number of infected migrants per day. For targeted parameters where data were not available from the published literature, a ±30% variation approach was applied to a one-way extreme value sensitivity analysis. We assessed the effect of these parameters on the number of infected cases before the easing of phase 1 restrictions on 3 May 2020.

### 2.5. Intervention Scenarios

Once the model had been validated, it was then used to predict the daily reported cases, and evaluate the impacts of the interventions on controlling the disease. We compared various scenarios, as follows:

A. The incidence of COVID-19 cases without the emergency decree (using the contact matrix of Thai setting with approximately ten contacts per day based on the contact rate from a previous study [27])
B. Easing the curfew on 15 June 2020 [28,29] (assuming that the contact rate among the population increased by 100% compared with the rate during the curfew period (approximately two contacts per day))
C. Easing the curfew on 15 June 2020 and easing of restriction phase 5 (high-risk businesses or activities were allowed to reopen) on 1 July 2020 [10] (assuming that the contact rate among the population increased by 300% compared with the rate during the curfew periods).
D. Easing the curfew on 15 June 2020, easing of restriction phase 5, and international travel ban [30] (assuming that ten infected migrants per day were taken into account when calculating the transmission rate and that the contact rate among the population increased by 300% compared with the rate during the curfew periods).
E. Easing the curfew on 15 June 2020 and easing of restriction phase 5, but hand washing and face mask wearing were implemented (assuming that the transmission rate was reduced by 10% [4] after the easing of restriction phase 5).
F. Easing the curfew on 15 June 2020 and easing of restriction phase 5 but the population continues to practice social distancing and work from home (assuming that the contact rate among the population reduced by 50% [31] compared with the easing of restriction phase 5).

## 3. Results

**Figure 3:**
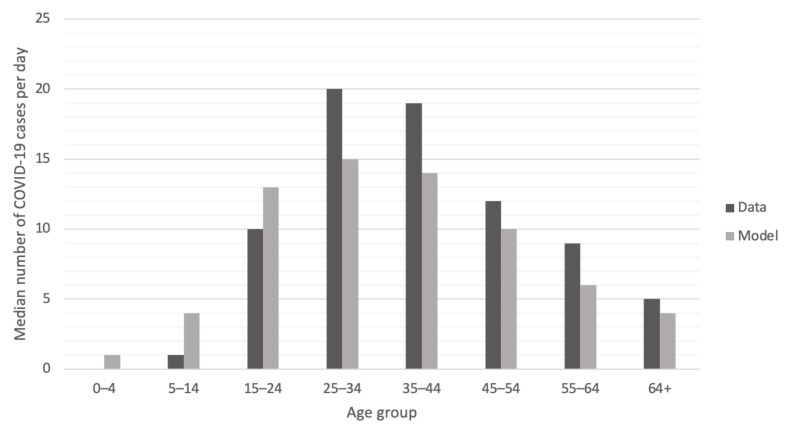
The median number of COVID-19 cases per day by age group by the observed and modeled data (dark gray: observed data, light gray: model projection).

During the early phase of the epidemic, in January 2020, Rt was estimated to be 2.68 (95% CrI: 2.47–2.89) as shown in the Supplementary Materials Figure, S2. Rt gradually decreased (to less than 2) during February and March. After the government declared the emergency decree and curfew, Rt rapidly decreased to 0.73 (95% CrI: 0.53–0.93) by the end of May. The age-specific observed and predicted incidences are shown in Figure 3. We found that the highest number of cases among those aged between 25 and 34 years occurred in March, both in the model and the actual data. A small number of cases were found in the younger age groups.

**Figure 4:**
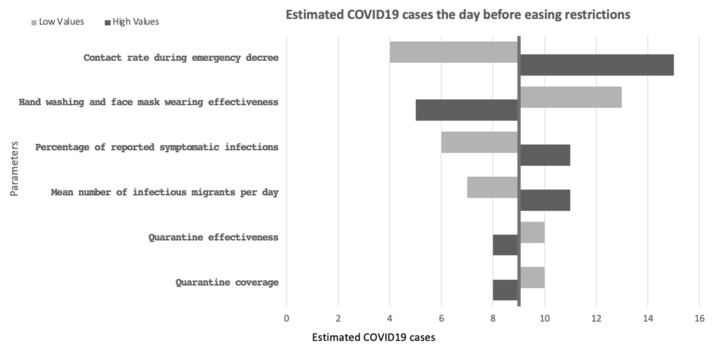
A tornado plot representing the estimated number of COVID-19 cases the day before restrictions were eased, by a one-way sensitivity analysis, ±30% variation approach (dark gray: high values; light gray: low values).

The one-way sensitivity analysis showed that the contact rate per day after the Thai government declared the emergency decree and before the easing of each restriction phase (range: 1–2 contacts per day) mainly affected the daily reported cases per day in the country followed by hand washing and face mask wearing effective (range: 14–26%), the percentage of all symptomatic infections that were reported (range: 14–26%) and mean number of infectious migrants per day (range: 0.0014–0.0026 per day) as shown in Figure 4. Quarantine effectiveness and coverage had the least effect on the number of cases.

**Figure 5:**
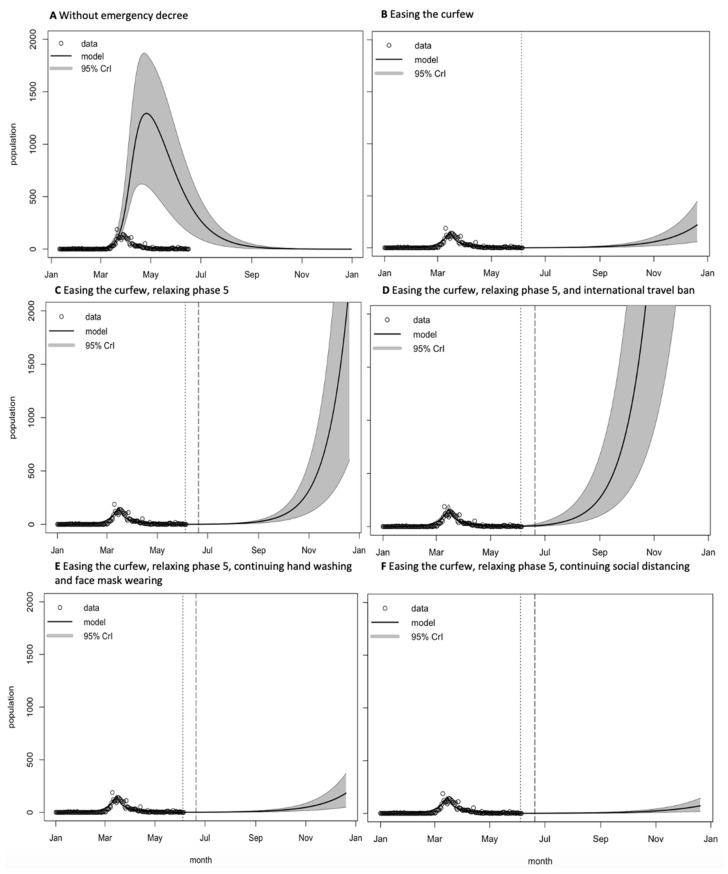
Predictions of COVID-19 incidence in Thailand under different scenarios. (**A**) Without an emergency decree; (**B**) easing the curfew on 15 June 2020, allowing social activities, high-risk businesses remain closed; (**C**) easing the curfew, relaxing phase 5 on 1 July 2020 (high-risk business or activities allowed to reopen); (**D**) easing the curfew, relaxing phase 5, and relaxing the international travel ban, (**E**) easing the curfew, relaxing phase 5, continuing hand washing and wearing of face masks; (**F**) easing the curfew, relaxing phase 5, continuing to practice social distancing (dotted line: easing the curfew on 15 June 2020; dashed line: relaxing phase 5 on 1 July 2020).

An analysis of the interventions is shown in Figure 5. If the government had not declared an emergency decree, the highest incidence of cases may have reached 1232 cases per day (95% CrI: 562–1812) by the end of April, as shown in Figure 5A. Easing the curfew and allowing social activities, but keeping high-risk businesses closed, may have increased the number of contacts per day among the population. It is likely that a second wave will emerge towards the end of 2020, with approximately 140 cases per day (Figure 5B).

Reopening high-risk businesses and allowing social activities (Scenario C) may increase the chances of a second wave occurring sooner than the previous scenario (Scenario B). The prediction of cases in Scenario C is more than one hundred cases per day by the end of the year, as shown in Figure 5C. Under Scenario D, we assumed that the mean number of infected migrants per day would be ten cases, after the easing of restrictions in the country. The model predicted the highest second wave with Scenario D compared with the other scenarios in October (Figure 5D), with a projected number of cases of more than a thousand cases per day. However, a way to reduce the size of the second wave would be to implement hand washing and the wearing of face masks among the population (Scenario E, Figure 5E). We predicted that the second wave under this scenario would be greater than with social distancing after the easing of restrictions (Figure 5F). A social distancing campaign, including working from home and avoiding being in crowded places (Scenario F, Figure 5F), is another way to reduce the risk of the second wave. The number of cases predicted to occur under this scenario was less than 45 cases per day. 

## 4. Discussion

Many mathematical models have been used to predict COVID-19 incidence and evaluate the impact of various interventions on both regional and global scales [6,17,18]. A previous study showed that predictions made using more complex models may not be more reliable compared with those made using a simpler model for COVID-19 [32]. Here, we applied a simple, compartmental deterministic model to evaluate the epidemiological properties of COVID-19 in Thailand. With few pharmaceutical products available, the practicing of NPIs is a key strategy for mitigating the spread of disease, including both personal prevention and epidemiological control, such as physical distancing, travel bans, wearing face masks, and wearing eye protection. The epidemiological patterns in each country are similar; however, the peaks of the disease burden in each country have differed according to their public health policies, human behavior, and culture. The Thai government established the Center for COVID-19 Situation Administration (CCSA) [2] to control this emerging disease through the implementation of nationwide health policies. Several policies were announced and implemented in Thailand, in various phases. The strictest policies, including a curfew, closing both high-risk and low-risk businesses, banning international travel, and working from home, were implemented during March, when the highest number of cases was reported. However, these strict policies were eased after case numbers declined.

Our estimation of Rt was consistent with that reported by WHO [33], with R0 reported to be 2 to 2.5, whereas our model estimated Rt to be about 2.68 (95% CrI: 2.47–2.89) during the early epidemic in Thailand. Our model could capture the effective interventions that the Thai government implemented during the early part of the epidemic, such as contact tracing, isolation, and quarantine [8], where the Rt was reduced accordingly (shown in the Supplementary Materials, Figure S2). An Rt of less than 1 is associated with the end-phase of an epidemic, i.e., decreasing disease incidence. This Rt threshold, therefore, has been used as a target for evaluating disease mitigation policies. Regarding the model’s projections, the Rt was estimated to be 0.73 (95% CrI: 0.53–0.93) during April and May, indicating the policies implemented were effective and were mitigating the disease burden in Thailand. The reduction of Rt to less than 1 after Thailand implemented strict intervention strategies has also been reported elsewhere [12,13]. Our SEIR model was able to accurately depict COVID-19 cases in Thailand, stratified by age. The working-age population (approximately 25 to 44 years) was most affected by the epidemic.

The daily incidence of COVID-19 was principally affected by the contact rate, hand washing, and face mask wearing effective during the period the emergency decree was in effect. Our sensitivity analysis results can pinpoint where an intervention is needed. The congruent reduction in contact rates between infected individuals (either symptomatic or asymptomatic) and susceptible individuals would effectively minimize the incidence of disease [34]. Interventions, such as physical distancing, working from home, limiting the number of people entering an enclosed place, and wearing a face mask, should be recommended to maintain the low contact rate in the country. While social distancing may show the biggest impact in the Thai setting, however this might not be the case in a different setting or different time frame. Several studies showed the similar benefits with our results of hand washing and face mask wearing against the transmission of COVID-19 [35,36,37]. In addition, the percentage of reported symptomatic and asymptomatic infections can influence the incidence. Approximately 17.9% of infected individuals are asymptomatic [38]. Hence, the detection of both symptomatic and asymptomatic individuals is a key strategy for minimizing this risk. Contact tracing and proactive testing in disease hotspots are effective activities for finding asymptomatic individuals who reside in a target area [16]. Additionally, the number of imported cases can affect the magnitude of an outbreak [39]. In Thailand, the number of confirmed cases was strongly associated with the number of tourists and their activities [40]. Therefore, interventions such as travel bans, closing the border, and the 14-day state or alternative quarantine for international travellers should be considered.

After declaring the state of emergency decree and implementing nationwide interventions, the contact rate in the Thai population decreased by 86% (95% CrI: 85–87%). The Rt also decreased by 60% and dropped below 1. This huge reduction in the Rt resulted from the implementation of a combination of several interventions. Social distancing alone can reduce the contact and infection rates of influenza by 50% and 23%, respectively [31]. During the COVID-19 epidemic in Wuhan, China, social distancing reduced the number of COVID-19 infections by 92% (interquartile range (IQR): 66–97) compared with no intervention [14]. This highly effective social distancing can be explained by the complete city lockdown and full restrictions imposed during the early epidemic in Wuhan. The implementation of social distancing in China during the outbreak was sufficient to control COVID-19 [41]. Our study showed that social distancing in Thailand was less effective because a complete city lockdown was not implemented. Therefore, a combination of intervention strategies is needed in Thailand to reach the same level of disease control.

Our model showed that a huge outbreak would have been expected had the emergency decree not been declared. Both confirmed cases and the positive detection rate in Thailand significantly declined in line with the stringency of the emergency decree imposed by the Thai government [9]. The city lockdown strategy was shown to be an effective intervention [42]. Here, we projected the expected number of COVID-19 cases in Thailand under several possible scenarios. A new surge in COVID-19 incidence will occur after the easing of each of the intervention strategies. However, the timing and magnitude of a new wave will depend on the maintenance of strictness of any interventions. It is inevitable that a new wave will occur in each country after their interventions and restrictions are lifted [16,19]. In some cases, for example Iran, the second wave may be more severe than the first wave [43]. Strategies suggested to reduce the magnitude of any new wave include social distancing, case isolation, quarantining of contacts, and increasing the level of proactive testing [19,44].

This study has some limitations. The model did not incorporate data relating to changes in behaviour, such as the frequency of wearing face masks, changing social contact patterns during the epidemic, and people’s mobility; such data are very limited. Obtaining data around migrant movements, especially illegal importation, is particularly challenging. In addition, some public health regulations, such as the level of testing, test confirmation procedures, delays in reporting etc., may vary throughout the phases of the pandemic and by the level of healthcare settings. The model simply assumed static values combined with reporting rates that varied by disease severity only. Even the case data from early in the first wave of the epidemic was limited, which might affect the estimation and prediction of parameter estimates, although the Rt and second-wave prediction results here are in agreement with other modeling studies conducted in Thailand [12,13,45] and other countries [16,43]. The compartmental model we developed assumed behavioral homogeneity among the entire population. This deterministic model does not allow the tracking of individuals to reflect performance measures of interventions, such as contact tracing, or the possible consequence of super-spreading events. This work provides some preliminary outputs in terms of disease projections and the evaluation of current interventions implemented in the Thai setting.

## 5. Conclusions

The first phase of COVID-19 incidence in Thailand was evaluated using an SEIR model. The contact rate, the percentage of reported symptomatic infections, the mean number of infectious migrants per day, and the practicing of NPIs were all found to play an important role in determining the magnitude of the incidence. Interventions aimed at reducing the first three of these parameters will assure the mitigation of any second wave. Our study suggested that public health policies, including the announcement of an emergency decree, physical distancing, contact tracing, travel bans, and wearing face masks, can help to prepare for and prevent a second surge in incidence. More stringent interventions have more preventive power than less stringent interventions. Therefore, the application of each intervention to a national population must be critically justified according to its cost-effectiveness. We believe that these results are generalizable and can be applied to other countries while pharmaceutical products remain under development.

## Data Availability

Data available in a publicly accessible repository that does not issue DOIs. Publicly available datasets were analyzed in this study. This data can be found here: https://covid19.ddc.moph.go.th/en.

## Supplementary Materials

The following are available online at https://www.mdpi.com/2079-7737/10/2/80/s1, Table S1: Parameter table for our COVID-19 model; Figure S1: Posterior distributions from the COVID-19 model; Figure S2: The effective reproductive number (Rt) of COVID-19 with 95% CrI in Thailand from January to May 2020; Figure S3: Reported COVID-19 cases who recovered and the fitted COVID-19 model with 95% CrI between January and August 2020; Figure: S4 Reported COVID-19 deaths and the fitted COVID-19 model with 95% CrI between January and August 2020.
