# Supplementary material for: Evaluating the Impact of Intervention Strategies on the First Wave and Predicting the Second Wave of COVID-19 in Thailand: A Mathematical Modeling Study
Source: Biology (Basel). 2021 Jan 22;10(2):80. doi: 10.3390/biology10020080 (PMC7911628; doi:10.3390/biology10020080)
Supplement: Supplementary file 1 [file biology-10-00080-s001.pdf]

Supplementary Files

Ordinary differential equations (ODEs) were used to predict COVID-19 infection dynamics in the Thai population. $N_j(t)$ is the number of people of the jth age group at time, t, and fr_j is the fertility rate in females aged j years. The number of newborn babies at any time [birth(t)] is shown as follows:

$$\text{birth}(t) = \sum_j fr_j \cdot N_j\ (t) \tag{1}$$

Mortality among the population [$\text{death}_j(t)$] was calculated from the age-specific mortality rate dr_j:

$$\text{death}_j(t) = dr_j \cdot N_j(t) \tag{2}$$

Ageing is defined as the rate at which individuals move to the next age group, also represented as at a rate $\frac{1}{\text{age.diff}}$ per year, where age.diff represents the difference between two age classes. Therefore, the matrix equation for individual dynamics was as follows:

$$\text{aging}_j(t) = \begin{bmatrix} -\text{aging}_1 & 0 & 0 & \cdots & 0 \\ \text{aging}_1 & -\text{aging}_2 & 0 & \cdots & 0 \\ 0 & \text{aging}_2 & -\text{aging}_3 & \cdots & 0 \\ \vdots & \vdots & \vdots & \ddots & \vdots \\ 0 & \cdots & \cdots & \text{aging}_7 & -\text{aging}_8 \end{bmatrix} \cdot \begin{bmatrix} N_1 \\ N_2 \\ N_3 \\ \vdots \\ N_8 \end{bmatrix} + \text{birth}(t) - \text{death}_j(t) \tag{3}$$

All parameters included in the model are shown in Table S1.

Table 1. Parameter table for the COVID-19 model.

Parameter	Symbol	Value (95% Credible Interval)	Source/ Reference
Population parameters			
The size of the population in Thailand	N_j	The Thai population stratified by age	Census data [1]
Fertility and mortality rates stratified by age	fr_j, dr_j	Fertility and mortality rates stratified by age	Census data and public health statistics [2]
Contact rate by age per day	contact_j	Average contact rate per day was 10 contacts	Meeyai A, et al. 2015 [3]
Contact rate by age per day after Thailand declared the emergency decree and before the easing of each restriction phase	$\text{contact}_j \times 0.155$ $(0.145 - 0.165)$	Average contact rate per day was 1.55 (1.45–1.65) contacts	Estimated by the Bayesian Markov Chain Monte Carlo (MCMC) method
Mean household size	Household_{si}	3 people	Household data [4]
Transmission parameters			
Transition rate of exposed individuals to the infected compartment (the mean incubation period was 5.2 (4–7) days)	σ	1/5.2	Qun Li et al, 2020 [5]
Transition rate of asymptomatic individuals to the recovery compartment	γ_A	0.2	Zou L et al, 2020 [6]
Transition rate of symptomatic individuals to the recovery compartment	γ_S	0.10526	Chen J, et al., 2020 [7] and WHO mission to China Report

Parameter	Symbol	Value (95% Credible Interval)	Source/ Reference
Transition rate of quarantined infected individuals to the recovery compartment	γ_q	0.11624	Tang B et al., 2020 [8]
Pre-symptomatic period	$\frac{1}{\eta}$	4 days	Gandhi M et al, 2020 [9]
Proportion of infected person who is asymptomatic	Asm_p	0.179 (0.155-0.202)	Mizumoto K et al, 2020 [10]
Relative infectiousness during the incubation phase	ρ	10%	Jing Q-L et al, 2020 [11]
Infectivity rate of COVID-19 infections	$magnit$	0.041 (0.0407-0.0414)	Estimated by the Bayesian Markov Chain Monte Carlo (MCMC) method
Effectiveness of quarantine	Q_{eff}	50%	Assumed
Effectiveness of hand washing and wearing face masks	$hand_{eff}$	20%	Doung-ngern, P., et al.,2020 [12] (noted that the coverage was taken into account in this parameter)
Effectiveness of isolation	$Isolate_{eff}$	35%	Kucharski, A. J., et al., 2020 [12]
Mean number of infected migrants per day	$importatio$	0.001 (0.0001-0.004) cases per day	Estimated by the Bayesian Markov Chain Monte Carlo (MCMC) method
Quarantine coverage	Q_{cov}	50%	Assumed
Duration of quarantine	$\frac{1}{Q_{day}}$	14 days	MoPH, 2020 [13]
Case fatality stratified by age	$case.fatal_j$	Age 0-4 = 0.007 (0.001-0.033)<br>Age 5-14 = 0.006 (0.001-0.025)<br>Age 15-24 = 0.006 (0.001-0.024)<br>Age 25-34 = 0.006 (0.001-0.026)<br>Age 35-44 = 0.006 (0.001-0.024)<br>Age 45-54 = 0.023 (0.006-0.054)<br>Age 55-64 = 0.024 (0.006-0.062)<br>Age more than 65 = 0.138 (0.082-0.209)	Estimated by the Bayesian Markov Chain Monte Carlo (MCMC) method
Percentage of all reported asymptomatic infections	Asm_r	2.1% (range 1.1-3.1%)	Estimated by the Bayesian Markov Chain Monte Carlo (MCMC) method

Parameter	Symbol	Value (95% Credible Interval)	Source/ Reference
Percentage of all reported symptomatic infections	Sm_r	20% (range 10-30%)	Assumed

The differential equations of the COVID-19 model in Thailand are as follows:

$$\frac{dS_j(t)}{dt} = birth(t) - \lambda\, S_j(t) - Q_{rate}S_j(t) + \frac{1}{Q_{day}}QS_j(t) + aging_j(t) - death_j(t) \tag{4}$$

$$\frac{dE_j(t)}{dt} = \lambda\, S_j(t) - \sigma E_j(t) - Q_{rate}E_j(t) + \frac{1}{Q_{day}}QE_j(t) + aging_j(t) - death_j(t) \tag{5}$$

$$\frac{dAsym_j(t)}{dt} = (Asm_p)\sigma \mathrm{E_j}(t) - \gamma_A Asym_j(t) - Q_{rate}Asym_j(t) + \frac{1}{Q_{day}}QA_j(t) + aging_j(t) - death_j(t) \tag{6}$$

$$\frac{dPsym_j(t)}{dt} = (1 - Asm_p)\sigma E_j(t) - \eta Psym_j(t) - Q_{rate}Psym_j(t) + \frac{1}{Q_{day}}QP_j(t) + aging_j(t) - death_j(t) \tag{7}$$

$$\frac{dSym_j(t)}{dt} = \eta Psym_j(t) + \eta QP_j(t) - \gamma_S Sym_j(t) + aging_j(t) - death_j(t) \tag{8}$$

$$\frac{dR_j(t)}{dt} = \gamma_A Asym_j(t) + \gamma_S Sym_j(t) + \gamma_q QA_j(t) + aging_j(t) - death_j(t) \tag{9}$$

$$\frac{dQS_j(t)}{dt} = Q_{rate}S_j(t) - \frac{1}{Q_{day}}QS_j(t) - \lambda q QS_j(t) + aging_j(t) - death_j(t) \tag{10}$$

$$\frac{dQE_j(t)}{dt} = Q_{rate}E_j(t) - \frac{1}{Q_{day}}QE_j(t) + \lambda q QS_j(t) - \sigma QE_j(t) + aging_j(t) - death_j(t) \tag{11}$$

$$\frac{dQA_j(t)}{dt} = Q_{rate}Asym_j(t) - \frac{1}{Q_{day}}QA_j(t) + (Asm_p)\sigma QE_j(t) - \gamma_q QA_j(t) + aging_j(t) - death_j(t) \tag{12}$$

$$\frac{dQP_j(t)}{dt} = Q_{rate}Psym_j(t) - \frac{1}{Q_{day}}QP_j(t) + (1 - Asm_p)\sigma QE_j(t) - \eta QP_j(t) + aging_j(t) - death_j(t) \tag{13}$$

Force of infection of natural diseases

$$\lambda_j = \beta_j \times \frac{(\rho E_j(t) + Asym_j(t) + Psym_j(t) + Isolate_{eff}.Sym_j(t) + importation)}{Ntotal_j} \tag{14}$$

$$\beta_j = (1 - hand_{eff}) \times magnit \times \mathrm{contact_j} \times Emergency.Decree.function \tag{15}$$

Force of infection of under quarantine

$$\lambda_{q,j} = \beta_{q,j} \times \frac{(\rho E_j(t) + Asym_j(t) + Psym_j(t) + Isolate_{eff}.Sym_j(t) + importation)}{Ntotal_j} \tag{16}$$

$$\beta_{q,j} = (1 - hand_{eff}) \times magnit \times \left(1 - Q_{eff}\right) \times contact_{home,j} \times Emergency.Decree.function \tag{17}$$

$$Q_{rate,j} = \left(\frac{(Asm_r(Asym_j(t) + Psym_j(t)) + Sm_r(Sym_j(t)) \times Household_{size}}{Ntotal_j}\right) \times Q_{cov} \tag{18}$$

Note: the j indicated to each stratified age group

Where,

$\frac{dS_j(t)}{dt}$ denotes the rate of change in the number of susceptible individuals at time t.

$\frac{dE_j(t)}{dt}$ denotes the rate of change in the number of exposed individuals at time t.

$\frac{dAsym_j(t)}{dt}$ denotes the rate of change in the number of asymptomatic individuals infected at time t.

$\frac{dPsym_j(t)}{dt}$ denotes the rate of change in the number of pre-symptomatic infected individuals at time t.

$\frac{dSym_j(t)}{dt}$ denotes the rate of change in the number of symptomatic infected individuals at time t.

$\frac{dR_j(t)}{dt}$ denotes the rate of change in the number of recovered individuals at time t.

$\frac{dQS_j(t)}{dt}$ denotes the rate of change in the number of quarantine susceptible individuals at time t.

$\frac{dQE_j(t)}{dt}$ denotes the rate of change in the number of quarantine exposed individuals at time t.

$\frac{dQA_j(t)}{dt}$ denotes the rate of change in the number of quarantine asymptomatic individuals at time t.

$\frac{dQP_j(t)}{dt}$ denotes the rate of change in the number of quarantine pre-symptomatic individuals at time t.

The number of cumulative hospitalized cases ($\mathrm{CumHos_j}$) was calculated by numerical integration of exposed cases ($\mathrm{E_j}$), quarantine exposed ($\mathrm{QE_j}$), and the transition rate of exposed individuals to the infected class (σ) as follows:

$$\mathrm{CumHos_j} = \int_{\mathrm{t}}^{\mathrm{t+1}} \left(Asm_r\left(Asm_p\right)\sigma(\mathrm{E_j} + \mathrm{QE_j}) + Sm_r\left(1 - Asm_p\right)\sigma(\mathrm{E_j} + \mathrm{QE_j})\right)\mathrm{dt} \tag{19}$$

The number of cumulative recovery cases ($\mathrm{CumRe_j}$) was calculated by numerical integration of asymptomatic cases ($\mathrm{Asym_j}$), symptomatic ($\mathrm{Sym_j}$), quarantine asymptomatic infected ($\mathrm{QA_j}$), and the transition rate of asymptomatic or symptomatic individuals to the recovery class (γ), as follows:

$$\mathrm{CumRe_j} = \int_{\mathrm{t}}^{\mathrm{t+1}} \left(Asm_r\left(\gamma_{\mathrm{A}}\mathrm{Asym_j}\right) + Asm_r\left(\gamma_{\mathrm{q}}\mathrm{QA_j}\right) + Sm_r\left(\gamma_{\mathrm{S}}\mathrm{Sym_j}\right)\right) \mathrm{dt} \tag{20}$$

The number of cumulative case fatality ($\mathrm{CumFatal_j}$) was calculated by numerical integration of exposed cases ($\mathrm{E_j}$), quarantine exposed ($\mathrm{QE_j}$), and the transition rate of exposed individuals to the infected class (σ), and case fatality proportion ($case.fatal_j$), as follows:

$$\mathrm{CumFatal_j} = \int_{\mathrm{t}}^{\mathrm{t+1}} \Big(case.fatal_j \Big(Asm_r\left(Asm_p\right)\sigma\left(E_j + EQ_j\right) + Sm_r\left(1 - Asm_p\right)\sigma\left(E_j + EQ_j\right)\Big)\Big)\,\mathrm{dt} \tag{21}$$

The Bayesian framework

Bayesian inference of the COVID-19 infection model provided a framework for estimating parametric uncertainty in terms of probabilistic distributions and allowing a direct quantification of parameter uncertainty.

Bayes' theorem states that the best estimate (posterior uncertainty $p(\theta|y)$) for a parameter vector θ given data y is given by:

$$p(\theta|y) = \frac{p(\theta)p(y|\theta)}{p(y)} \tag{22}$$

Here, $p(\theta)$ is the prior information and, $\frac{p(y|\theta)}{p(y)}$ is the likelihood ratio. Markov Chain Monte Carlo (MCMC) algorithms were applied to approximate these distributions, which used a sampling scheme to estimate the posterior distribution [14,15].

Prior distribution

Uniform distribution was applied to all prior parameter values, given that little information about these parameters has been measured or reported. The minimum and maximum values were initially determined to constrain the feasible range of a parameter, thereafter it was narrowed down during an iterative model fitting procedure.

Likelihood function

The likelihood was defined as the product of likelihood terms for each data point. The data arise from the daily reported incidence, recovery cases, and deaths and are linked to the summation of those expected values by age via a Poisson distribution. The log-likelihood (used as the target in the MCMC algorithm) is:

Likelihood function of the developed COVID-19 dynamic model is,

$$LL_j = \sum_j \left(\sum_t \log \left(\frac{R^{\theta_c} \exp(-R)}{\theta_c!} \right) \right) \tag{23}$$

Where θ_c is the number of daily reported incidences, recovery cases, and deaths aged j at time t; R is the expected number of daily reported incidences, recovery cases, and deaths from the model prediction at each age class j^{th} and time t.

Posterior estimation

Differential Evolution MCMCzs (DE-MCzs) were applied to estimate the parameter posterior distributions. Observations were fitted using the Markov Chain methods of sampling [16] implemented in the Bayesian Tools R package, which is generally applied for numerical problems. Differential Evolution Markov Chain (DE-MC) is an adaptive MCMC algorithm. This algorithm can optimize the runtime using multiple chains in parallel and presenting them. DE-MCzs combine characteristics of conventional MCMC methods with the ideas of differential evolution optimization algorithms by making use of the full joint density function and (independent) proposal distributions for each of the variables. Posterior parameter distributions from the COVID-19 dynamic model are illustrated in Figure S1.

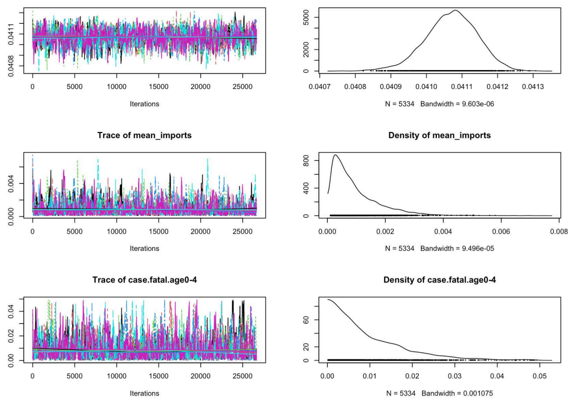

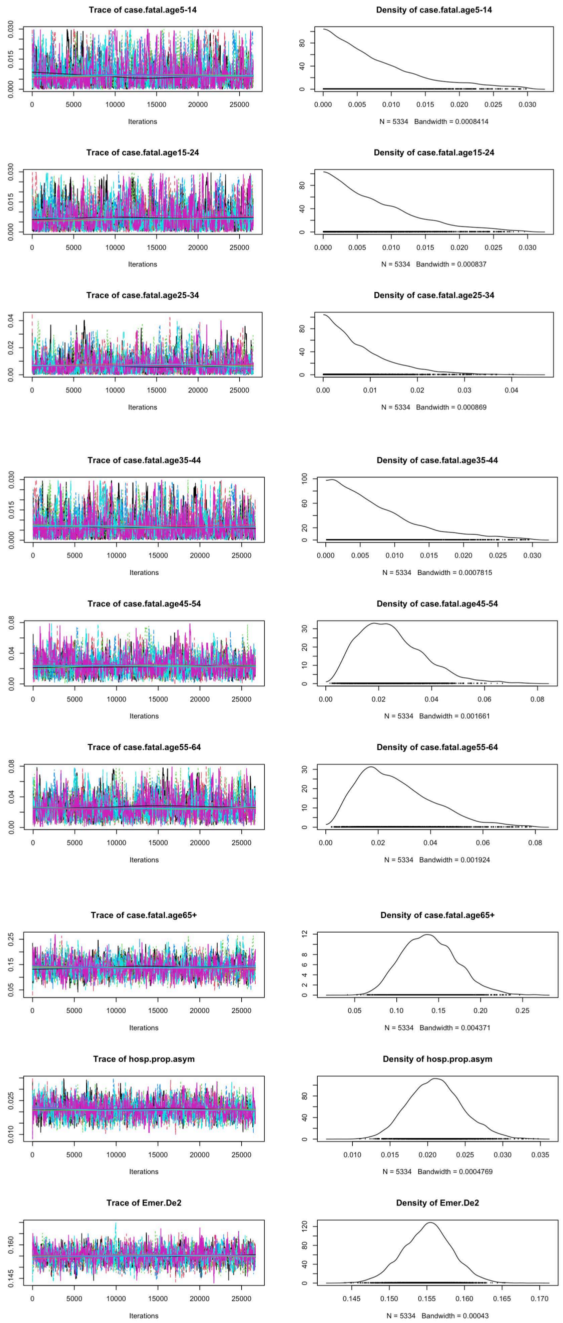

Figure S1. Posterior parameter distributions of the COVID-19 dynamic model, with each row corresponding to a separate parameter, the left-hand column contains traces with 6-colour chains (dashed lines: actual traces, solid lines: trends) and the right-hand column contains the posterior distribution, corresponding to each parameter.

Estimation of the effective reproductive number (Rt) of COVID-19 in Thailand

The effective reproductive number (Rt) was calculated based on the average number of people who become infected by an infectious person, as in the following equation and Figure S2.

$$Rt = \frac{\sum_{t}^{j}(\sigma \times E_t)}{\sum_{t-\frac{1}{\gamma}}^{j}\left(\sigma \times E_{t-\frac{1}{\gamma}}\right)} \tag{24}$$

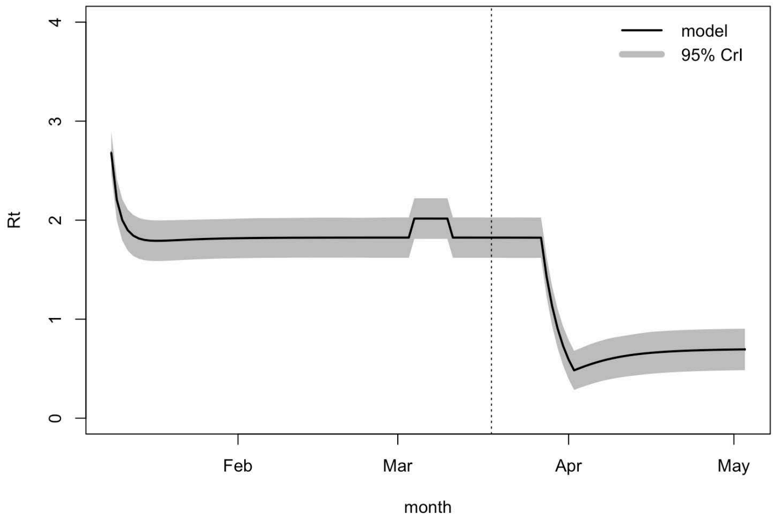

Figure S2. The effective reproductive number (Rt) of COVID-19 with 95% CrI in Thailand from January to May. (Vertical dotted line: the Thai government announced the emergency decree on March 26, 2020).

The model fitting results

The COVID-19 recovery cases per day between the data and the model are shown in Figure S3.

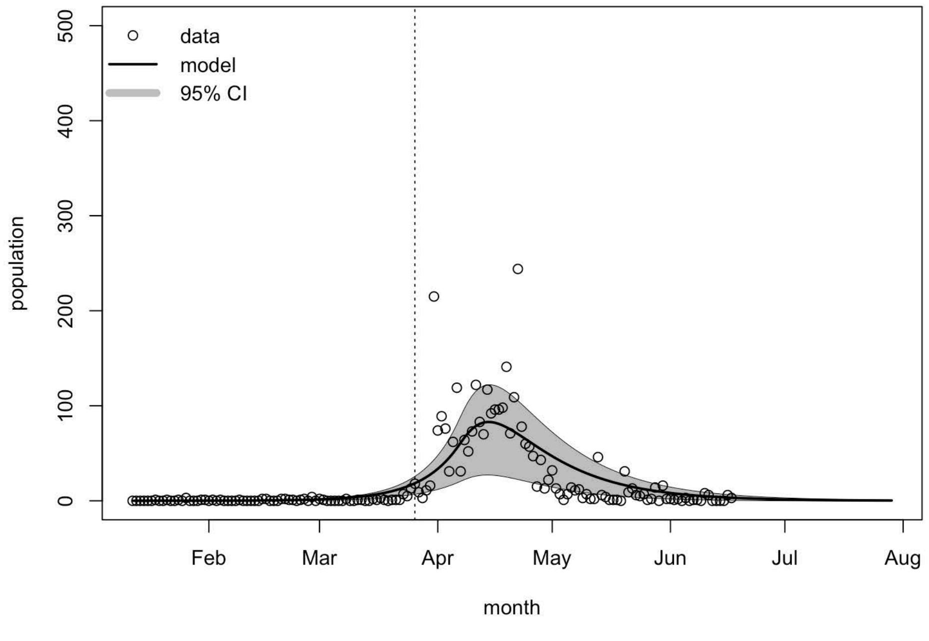

Figure S3. Reported COVID-19 recovery cases and the fitted COVID-19 model with 95% CrI between January and August 2020. (Vertical dotted line: the Thai government announced the emergency decree on March 26, 2020).

COVID-19 deaths per day between the data and the model are shown in Figure S4.

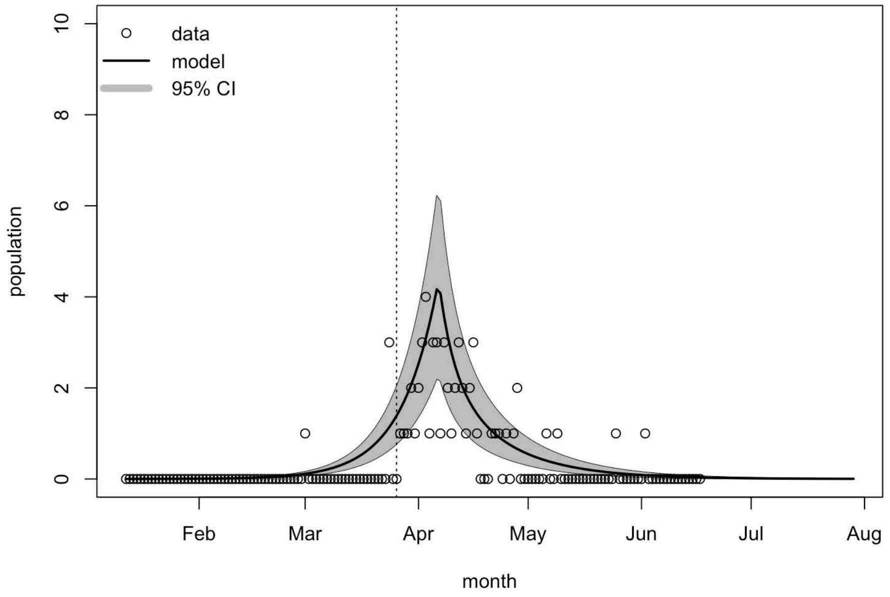

Figure S4. Reported COVID-19 deaths and the fitted COVID-19 model with 95% CrI between January and August 2020. (Vertical dotted line: the Thai government announced the emergency decree on March 26, 2020).

References:

1. BORA. Official statistic registration systems. Availabe online: https://stat.bora.dopa.go.th/stat/statnew/ statMenu/newStat/home.php (accessed on 9 December, 2020).
2. MoPH. Public Health Statistics, A.D.2018; 2018.
3. Meeyai, A.; Praditsitthikorn, N.; Kotirum, S.; Kulpeng, W.; Putthasri, W.; Cooper, B.S.; Teerawattananon, Y. Seasonal Influenza Vaccination for Children in Thailand: A Cost-Effectiveness Analysis. PLOS Medicine 2015, 12, e1001829, doi:10.1371/journal.pmed.1001829.
4. UNFPA. The State of Thailand's Population 2015, Features of Thai Families in the Era of Low Fertility and Longevity; the United Nations Population Fund Thailand and the Office of the National Economic and Social Development Board: Thailand, 2015.
5. Li, Q.; Guan, X.; Wu, P.; Wang, X.; Zhou, L.; Tong, Y.; Ren, R.; Leung, K.S.M.; Lau, E.H.Y.; Wong, J.Y.; et al. Early Transmission Dynamics in Wuhan, China, of Novel Coronavirus–Infected Pneumonia. New England Journal of Medicine **2020**, *382*, 1199–1207, doi:10.1056/NEJMoa2001316.
6. Zou, L.; Ruan, F.; Huang, M.; Liang, L.; Huang, H.; Hong, Z.; Yu, J.; Kang, M.; Song, Y.; Xia, J.; et al. SARS-CoV-2 Viral Load in Upper Respiratory Specimens of Infected Patients. New England Journal of Medicine **2020**, *382*, 1177–1179, doi:10.1056/NEJMc2001737.
7. Chen, J.; Qi, T.; Liu, L.; Ling, Y.; Qian, Z.; Li, T.; Li, F.; Xu, Q.; Zhang, Y.; Xu, S.; et al. Clinical progression of patients with COVID-19 in Shanghai, China. Journal of Infection 2020, 80, e1-e6, doi:https://doi.org/10.1016/j.jinf.2020.03.004.
8. Tang, B.; Wang, X.; Li, Q.; Bragazzi, N.L.; Tang, S.; Xiao, Y.; Wu, J. Estimation of the Transmission Risk of the 2019-nCoV and Its Implication for Public Health Interventions. J Clin Med **2020**, *9*, 462, doi:10.3390/jcm9020462.
9. Gandhi, M.; Yokoe, D.S.; Havlir, D.V. Asymptomatic Transmission, the Achilles' Heel of Current Strategies to Control Covid-19. New England Journal of Medicine **2020**, *382*, 2158–2160, doi:10.1056/NEJMe2009758.
10. Mizumoto, K.; Kagaya, K.; Zarebski, A.; Chowell, G. Estimating the asymptomatic proportion of coronavirus disease 2019 (COVID-19) cases on board the Diamond Princess cruise ship, Yokohama, Japan, 2020. Euro Surveill **2020**, *25*, 2000180, doi:10.2807/1560-7917.ES.2020.25.10.2000180.
11. Jing, Q.-L.; Liu, M.-J.; Zhang, Z.-B.; Fang, L.-Q.; Yuan, J.; Zhang, A.-R.; Dean, N.E.; Luo, L.; Ma, M.-M.; Longini, I.; et al. Household secondary attack rate of COVID-19 and associated determinants in Guangzhou, China: A retrospective cohort study. The Lancet Infectious Diseases 2020, 10.1016/S1473-3099(20)30471-0, doi:10.1016/S1473-3099(20)30471-0.
12. Kucharski, A.J.; Klepac, P.; Conlan, A.J.K.; Kissler, S.M.; Tang, M.L.; Fry, H.; Gog, J.R.; Edmunds, W.J.; Emery, J.C.; Medley, G.; et al. Effectiveness of isolation, testing, contact tracing, and physical distancing on reducing transmission of SARS-CoV-2 in different settings: A mathematical modelling study. The Lancet Infectious Diseases 2020, 10.1016/S1473-3099(20)30457-6, doi:10.1016/S1473-3099(20)30457-6.
13. MoPH. Covid-19 Situation Reports 2020.
14. Augustynczik, A.L.D.; Hartig, F.; Minunno, F.; Kahle, H.-P.; Diaconu, D.; Hanewinkel, M.; Yousefpour, R. Productivity of Fagus sylvatica under climate change – A Bayesian analysis of risk and uncertainty using the model 3-PG. Forest Ecology and Management **2017**, *401*, 192–206, doi:https://doi.org/10.1016/j.foreco.2017.06.061.

15. Hartig, F., Minunno, F., Paul, S.,. BayesianTools: General-Purpose MCMC and SMC Samplers and Tools for Bayesian Statistics. R package version. R package version 0.1.3. Availabe online: https://cran.r-project.org/web/packages/BayesianTools/ndex.html (accessed on 9 November,2020).
16. Ter Braak, C.J., Vrugt, J.A. Differential evolution Markov chain with snooker updater and fewer chains. Stat. Comput. **2008**, *18*, 435–446.

.
